# Contaminants in Foods of Animal Origin in Cameroon: A One Health Vision for Risk Management “*from Farm to Fork*”

**DOI:** 10.3389/fpubh.2017.00197

**Published:** 2017-09-04

**Authors:** Guy B. Pouokam, B. U. Saha Foudjo, Chi Samuel, Philomina Fankam Yamgai, A. Kamda Silapeux, Joel Taguemkam Sando, G. Fankam Atonde, Chiara Frazzoli

**Affiliations:** ^1^Laboratory of Food Safety, Biotechnology Center, University of Yaoundé 1, Yaoundé, Cameroon; ^2^Laboratory of Food Sciences and Metabolism, University of Yaoundé 1, Yaoundé, Cameroon; ^3^Virtual Resource Education Services, Douala, Cameroon; ^4^Department of Cardiovascular, Dysmetabolic and Aging-Associated Diseases, Istituto Superiore di Sanità, Rome, Italy

**Keywords:** contaminants, residues, One Health, risk management, toxicants, adult equivalent

## Abstract

Foods of animal origin represent an important share in the diet of Cameroonian populations. Cameroon is known to be a food basket in the west and central Africa sub-region, and an important supplier of foods on the international markets. In the meantime, food importation is continuously increasing to meet the high demand of a more westernized segment of the population. Cereals, fish, sea products, eggs, honey, shrimps, chicken, and feed ingredients are important share in the international trade of agricultural products. Few controls are made on the quality and safety of these products. Certain safety standards do exist but are still yet to be enforced. Inspections done so far by regulatory authorities are partial and do not cover important hazards that require laboratory analysis. The increasing awareness of population, the burden of new types of disease, as well as the recurrence of food scandals have recently launched a scientific and population debate on the contribution of foods items, especially those of animal origin, to the toxic exposure of food producing animals and humans. This paper critically reviews the occurrence of toxicants in most consumed foods of animal origin in Cameroon. This study included the most consumed food of animal origin, identified during the national household budget survey and contributing to 8.1% of the total diet of an individual. Data evaluated suggest an important contamination by toxic metals, mycotoxins, veterinary drugs’ residues, and pesticides. The current national legal framework is briefly analyzed to explore possible intervention measures in the frame of the One Health approach.

## Introduction

Cameroon is known to be the Africa in miniature. Situated at the heart of the central Africa region, the country is classified as a lower and middle income country (1). Food insecurity, malnutrition, and food intoxication remain heavy challenges. Health indicators are seen as an alert: life expectancy at birth has timidly increased in average, from 54 years (1985), to 56 years in 2015 (2). Exclusive breastfeeding is estimated at 20%. Prevalence of severe or moderate underweight and stunted children under 5 years are at 15 and 33%, respectively (3). According to the same references, known causes of deaths of under-5 children are: pneumonia 15%, malaria 12%, diarrhea 11%, HIV/AIDS 3%, and congenital diseases 2%. It remains important to note that up to 22% causes of infant deaths remain unidentified and unknown (2). These figures are subjects of hot discussions among health professionals and within the population. In addition, the emergence of non-communicable diseases (NCD), from cancer to hypertension, diabetes, and congenital malformations (4–6) throw some light on some risk factors. Hazards in foods of animal origin are gradually considered as public health threats in Africa: if inappropriately produced and handled, foods may be vectors of various toxic contaminants. For instance, the Yaoundé cancer diseases registry estimates the cancer incidence rate according to age group at 107 new cases per 100,000 inhabitants, with 42% male and 58% female (7). Contribution of food contaminants in the occurrence of these cases is still controversial. They however recognized that many causes of cancer have been so far underlined by international bodies, including eating of red meat and processed meats, consumption of low fiber diets, absence of breast feeding, obesity, increase of adult height, and practice of sedentary lifestyles (8).

Specific lifestyle risks factors like the decrease in physical activity, and consumption of energy-dense diets, associated with genetic predisposition, are also well-known criteria in the onset of metabolic syndrome and related comorbidities (obesity, diabetes, and cardiovascular diseases). On the other side, the limited success in reversing such morbidity cases by focusing uniquely on nutrition, exercise or drug therapies again fosters the hypothesis of a significant contribution from environmental factors like chemical pollutants. Because pre- and postnatal metabolic programming is largely dependent on endocrine homeostasis, endocrine disrupting chemicals are suggested to play a role as risk factors in the onset of metabolic syndrome (9): the burden of neoplastic and infectious diseases has been related to the rising environmental pollution (10), especially in economically developing countries.

The ongoing concerns in Cameroon underlines the need for collaborative research on perceptions, practices, and behaviors of actors at all levels of food chains, in order to identify diseases’ risk factors and their interplay in diseases appearance (11). This paper reviews some findings regarding contaminants in most consumed foods of animal origin in Cameroon. This gives a picture of the actual situation, as well as orientations to better investigate sources of contamination and assess population health risk.

## Most Consumed Foods of Animal Origins

Foods of animal origin constitute a significant share of the Cameroonian diet. Food consumption data have been estimated at national level during the second Cameroonian Household Budget Survey (HBS/ECAM II) in 2001 (12). The data are expressed for consumers only and per adult equivalent (AE). This survey revealed that consumption of animal products covered up to 8.1% of the total diet including fish (52 g/day per AE), beef, poultry and eggs (17 g/day per AE), and milk and dairy products (10 g/day per AE). In particular, smoked fish is the most consumed food of animal origin (22.4 g cooked/day per), followed by mackerel (18.3 g cooked/day) barrel fish (10.9 g cooked/day), poultry (9.81 g cooked/day), eggs (7.15 g cooked/day), and beef with bone (11.1 g cooked/day). Fresh water fish (12.4 g/day), sea fish (10.4 g/day), evaporated sweetened full-cream milk (7.2 g/day), and shrimps (1.3 g/day) are also consumed.

Milk and dairy products are also widely consumed in all 10 regions of the countries. Milk products are imported and also locally produced. In Northern and Eastern regions where animal breeding is the dominant agricultural activity, a lot of traditional milk and milky products are produced for daily consumption.

Honey for its properties is commonly used to replace sugar in various food preparations, especially during breakfast and during formulation of traditional medicine especially for infants.

With more than 250 ethnic groups, insects eating are ancient cultural habits for certain ethnic groups found in the south, center, east, and western region.

Because these foods from animal origin represent the most important source of animal proteins in the diet of an average individual, we reviewed some contaminations already reported in these food matrices. In this review, we also use a similar foods grouping to match the ones used by authorities for national household survey.

## Occurrence of Contaminants in Most Consumed Food Groups and Feeds

### Fish and Sea Foods (Mackerel, Barrel, Smoked Fish, Shrimps, Freshwater Fish)

The fishery sector plays an important role in Cameroon. It is a well appreciated source of affordable and accessible animal protein for a huge portion of the population. Cameroon produces many fish species, both from the industrial fleet and artisanal operators. Cameroon has been exporting fish products to the European Union market. The main export from Cameroon in 2010 was shrimp, it was banned because of insufficient hygiene conditions and inappropriate official control on products destined for the export market (13). An audit mission report from the EU food and veterinary office identified some lapses in official control (deliverance of health certificate, laboratory analyses, and water quality). Journalists from the “green news infos” reported findings by Ntaryike in 2016 (14) from a study done in the Douala coastal borders, on the high contamination of fish with mercury. Moreover, traditional methods for fish drying constitute additional sources of contamination. The national newspaper “Cameroon tribune” (15) reported that some local fishermen use toxic chemicals in order to improve their catch, while those who smoke fish use plastics and worn-out car tyres. Ahmed et al. (16) studied the influence of smoke and traditional drying on the quality of three fish species coming from the Lagdo Lake. All fish samples analyzed were found to be of poor microbiological quality. *Escherichia coli*, fecal streptococci, *Staphylococcus aureus*, sulfite-reducing clostridia, and molds were detected at levels above recommended standards.

Gimou et al. (12) estimated the average intake of some toxic metals by Yaounde population. Aluminum intake from fish was estimated to be in average at 11.4 µg/kg body weight/day, 0.155 µg/kg body weight/day was found for cadmium, and 0.963 µg/kg body weight/day for lead. The same study showed that fish was among the major contributing food to population exposure to aluminum, with boiled “dried and smoked fish and shrimps” representing up to 15% of the total exposure. Boiled fish “mackerel” accounted for 7% of cadmium exposure in the whole population. Fish was found to be the food group containing in average the highest quantity of total arsenic (1.20 mg/kg), therefore accounting for up to 71% of total arsenic exposure in the population; boiled mackerel fish alone constituted 33% of this exposure level, followed by smoked fish and shrimps 24%. Arsenic in fish is usually mainly present in the organic form, with limited toxicity effects. Moreover, in water bodies, the inorganic mercury is methylated to methylmercury (MeHg). This methylated form is the most toxic organic form which is capable to bioaccumulate in marine organisms, and biomagnifies through the entire food chain. Fish is by far the most important dietary vehicle of MeHg (17). The total amount of mercury was quantified only in the fish group, being one of the two food groups of outdoor meals that contribute to population exposure to MeHg (18). Smoked fish and shrimps represented 6% of lead exposure, and 6% of nickel exposure. Vanadium was also found to be present in fish products at a concentration of 0.167 mg/kg. Fish products were identified as the main vector of exposure of Yaounde population to vanadium with 43% for the total exposure share. The very first Cameroonian Total Diet Study (19) revealed that pesticides residues were not detected in fish products.

### Poultry

Poultry meat consumption accounted for 9.81 g/day/AE. With regard to the quality and safety of this product, the issue had been raised since the year 2000 and an important concern was the ban of the importation of frozen chicken. Nzouankeu et al. (20) evaluated the prevalence of pathogenic microorganism (*E. coli, Campylobacter*, and *Salmonella*) in imported frozen chickens. One hundred and fifty chickens were collected from eight retail markets in Yaoundé and were examined for the presence of these microorganisms, using standard bacteriological procedures. Out of the 150 chickens, 90% were contaminated with *Campylobacter* (68.9% *C. coli* and 31.1% *C. jejuni*). All the chickens showed the presence of *E. coli*. Among the 150 isolates obtained, 11.3% were enteropathogenic *E. coli*. Furthermore, 103 *Salmonella* strains were also discovered in 90 chickens. 45.6% of *Salmonella* Enteritidis and 28.1% *Salmonella* Hadar were found to be the most common serotypes present. Multiple contamination was found in 94.6% chickens, of which 83 (i.e., 55.3%) were concurrently contaminated with *Campylobacter, E. coli*, and *Salmonella*. Aflatoxin B1 has also been detected in gizzard and chicken muscle (21). In the second Cameroonian Total Diet Study, Gimou et al. (22) found out that poultry meat contains cadmium at a concentration of 0.019 mg/kg.

### Eggs

Average consumption of boiled eggs by the whole population is estimated to be 3.86 g/day (19). Other consumption methods like frying with other ingredients and swallowing whole eggs were not considered. Moundipa et al. (23), determined the presence of aflatoxin in eggs collected from different poultry farms, in different agro-ecological zones of the country, polled together to make one composite sample for laboratory analysis. 45.2% of the eggs were found to have detectable level of Aflatoxins (AFB_1_, AFB_2_, and AFM_1_). In addition, they found out that the forest zone had the highest toxin contamination. The level of cadmium has been estimated at 0.019 mg/kg (22).

### Beef and Pork Meat

Cooked beef with and without bone represents 11.1 and 8.61 g/day/AE, respectively. Meat inspection and control remains insufficient all over the country. The hygiene conditions of slaughter houses constitute crucial point to guarantee quality of the final product. In Cameroon, only two modern slaughter houses exist in Douala and Yaounde, others are traditional. A classification of traditional slaughterhouses and butcher shops based on microbiological characteristics of beef was conducted in the Northern part of Cameroon by Afnabi et al. (24). They collected 125 samples. Microbiological analyses showed significant contamination of carcasses in slaughterhouses, with average concentrations of 4.03 ± 0.8, 2.26 ± 0.8, 0.37 ± 0.55, and 2.2 ± 1.02 log cfu/cm^2^, respectively, for mesophilic aerobic bacteria, coagulase-positive staphylococci, anaerobic sulfur-reducing bacteria, and thermo tolerant coliforms. In a previous study, Afnabi et al. (25) administered questionnaires to a number of 469 assistant butchers, from 15 traditional slaughter houses in the area of the study. The objective was to evaluate their perception of basic rules of hygiene. Their conclusion was that, whatever the types of slaughterhouses (traditional) found in the northern part of Cameroon, the hygiene practices were mainly linked to the poor know—how and management of personnel, as well as during production (treatment process of carcasses).

Fonkem et al. (26) assessed the microbiological quality of a traditional dried meat called “Kilishi.” Seventy nine samples of Kilishi were collected at various selling points. The results showed that the quality of Kilishi was highly affected by the location of the production and the season. The total counts (colony-forming unit/gram) of bacterial, mold, and yeast were lower than recommended accepted limit, as well as the total viable bacterial counts of micro-organisms in meat at the point of consumption.

Pork meat consumption is rapidly growing among all classes in the society. It is eaten in certain regions of the country as traditional food, but its consumption is also urbanized. It is eaten in restaurant and out on the street as vended foods. Pork meat has become a major source of protein and fats. Yannick et al. (27) analyzed the bacteriological profile of pork meat prepared and sold along commercial streets of Nkwen and Bambili in the North-west region. Eleven (duplicate) pork samples were randomly collected and analyzed for bacteria. 100% of the pork meat samples confirmed the presence of bacterial pathogens: *S. aureus* (81.8%); followed by *Klebsiella pneumoniae* (72.7%), *Escherichia coli* (54.4%), *Salmonella* spp. (45.4%), *Proteus vulgaris* (27%), and *Shigella* spp. (9%). Djoulde et al. (28) carried out a study on street-vended meat samples purchased from street food sellers in five major towns from Soudano–Sahelian zone of northern Cameroon. The total aerobic microflora, *S. aureus, Bacillus cereus, Salmonella, Escherichia coli* type 01 non-0157:H + *Escherichia coli* strain, yeast, and molds were checked. The mean aerobic counts and *E. coli* in roasted beef meat, fried pork meat, and roasted chicken for all street-vended samples collected from mobile and stationary food sellers were not significantly different from one to another. However, all the counts were as much as the permitted level of count (3.0 log10/g) for cooked foods. Based on the relatively low bacterial counts, the quality and safety of street-vended meat products analyzed in this study was considered to be acceptable. Meats from slaughtering houses are inspected by certified inspectors before being sent to the market. Unfortunately, there is no regulation or standard specifying the parameters that have to be checked and verified; currently, inspection consists of physical checking for any abnormalities and very few laboratory test (e.g., temperature, pH). It is therefore difficult to gain information on toxicological risk. Practices at risks are however well known, such as improper use of veterinary drugs for animal treatment, feed quality, and bad hygienic conditions from slaughtering to markets points. Meat transport is an important point for all sorts of contamination. No quality control is done once the meat is at the market.

### Milk and Dairy Products (Sweetened Full-Cream Milk, Industrial Yogurt)

**Figure 1:**
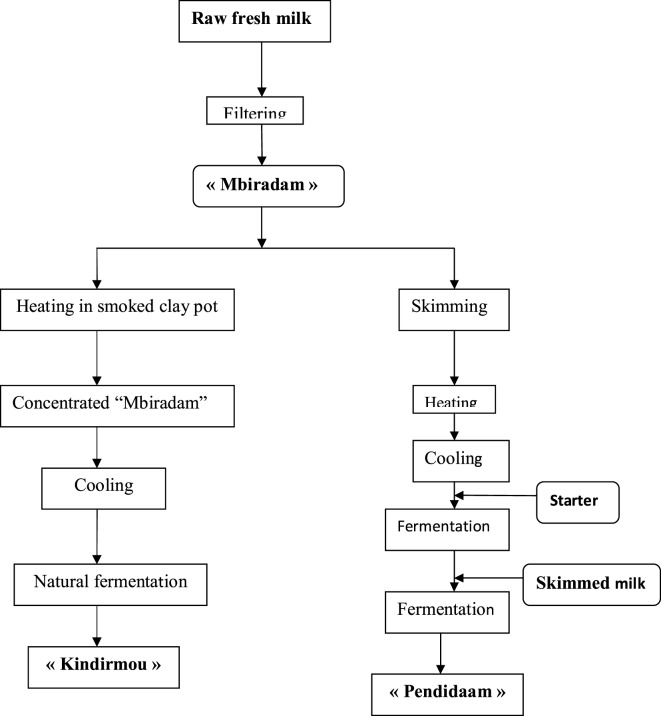
Artisanal process production of local milk “Kossam” (Mbiradam, Kindirmou, Pendidaam) by Djoulde et al. (30).

Milk and dairy products are consumed in different forms across the 10 regions of Cameroon: evaporated full-cream milk, powdered full-cream milk, and other local traditional forms such as *Kossam* (milk in peul language), *lebol* (traditional fermented milk), and *Kindirmou* (traditional butter). The northern regions of Cameroon (Far North, North, Adamaoua) are the main producers of milk. According to the processing type, the specific terms used are as follows: *Biraadam* for the raw, fresh, non-fermented, unskimmed milk; “*Kindirmu*” for thick milk, this is ordinary milk, heated and coagulated; “*Penndiidam*” for fermented milk made from skimmed “*Biraadam*,” heated and fermented and “*Dakéré*” for a mixture of fermented milk and cassava semolina; yogurt. There are two types of yogurt, i.e., the factory made yogurt and the semi-manufactured type marketed under the label Kossam (29). The traditional production process of “*kossam*” was described by Djoulde et al. (30) as in Figure 1.

Milk and dairy products are widely used for infant feeding, whereas the average consumption of milk products by the population according the mentioned total diet study is up to 7 g/day per adults. Dietary exposures to trace elements were calculated from these same studies. Milk and dairy products were found to be the food groups containing most calcium on average (4,161 mg/kg), the second for potassium (4,750 mg/kg). If these products are appreciated for their high nutritive content, they are also known as potential carriers of various contaminants. Production conditions and application of improper procedures during milking and processing greatly affect the quality and safety of these products (31). Local and traditional milk factory are most vulnerable to diverse and massive contamination with public health importance. “*Lebol*” and “*Kindirmou*” are two local dairy products mostly consumed in the northern part of the country. Edima et al. (29) investigated two production sites of these products in the Adamawa Region. Questionnaires were administered to farmers; additional observations and microbiology analyses were also carried out. Good hygienic practices for the essentials were not respected and ignored by producers. Both “*Lebol*” and “*Kindirmou*” products were contaminated with yeast/mold germs.

Moundipa et al. (23) indicated the amount of aflatoxin in milk. They detected aflatoxin M1 in 15.9% of cow raw milk at levels up to 0.525 µg/L. Levels of antibiotics residues contamination in raw milk were assessed in Ngaoundere (Adamawa Region). The veterinary doctor reported the main use of three antibiotics (oxytetracyclin, penicillin, and streptomycin) in cow health; 27% of milk samples collected in various farms of the locality was found to be contaminated with one or more antibiotic residues. Antibiotics of the beta-lactams and/or tetracycline families (penicillin, oxytetracyclin) were suspected to be possible sources of contamination for 53.85% of milk samples, while antibiotics residues of macrolide and/or aminoglycoside (streptomycin) were detected in 15.38% of the samples (32).

Aflatoxins are known to be toxics and have been proved to be a cause of human liver cancers. In high doses, they are also causes of deaths from aflatoxicosis (33). Aflatoxin M1 was found in milk (21) and can be transmitted to unborn baby through breast milk (34) and potentially cow milk. Cow’s milk in Africa is known to be a major food for young children. This stresses the importance of AFB1 monitoring in milk, dairy products, and in food products of animal origin as a whole. Moundipa et al. (23) detected aflatoxin metabolites in urine from children suffering from kwashiorkor and marasmic diseases (45.5%), and in the body fluids (sera) of 63.9% of primary liver cancers patients. However, the combination of these risk factors could not justify the increase in incidence and prevalence of malnutrition and cancer in Cameroon. As management measure, a cost-effective animal health-milk safety scheme should be established in the complex, multifaceted scenario of dairy production chain in Africa (35).

### Honey

Honey is a sugary substance, produced from the nectar of certain flowers by the worker bees. It is a complex mixture that can present large variations in their composition and characteristics depending on their botanical and geographical situation (36). The consumption of honey is constantly growing locally because of its high nutritional value and therapeutic claims in the treatment of various diseases. Yeast and spore forming microbes are useful indicators of the sanitary and commercial quality of honey. Cameroon is listed among the recognized non EU-countries which are allowed to export honey in the European Union (37). In the heart of the “Oku mountain” in the North-west region of Cameroon, the best honey in the world is produced: “The white Honey of Oku.” The Oku Mountain provides a unique ecosystem for the production of this honey. The Oku honey is one of the three African products to have received in 2013 the label “Geographical Protected Indication” by the African Organization for Intellectual Property. This calls for a more stringent residue monitoring plan for the analysis of antibiotics residues, sulfonamides, pesticides, and heavy metals to meet standards. In Cameroon, common practices for profit reasons are to dilute pure honey with a little amount of water before selling. This is believed to affect the quality of the products, and also it shelf-life.

The increasing numbers of consumer’s awareness on foods risks, coupled to trade globalization, are driving the honey markets. The global production worldwide is constantly increasing since 2000 (37). Honey can be polluted *via* different sources of contamination. In Cameroon, some concerns are related to the use of pesticides, antibiotics, and microorganisms. Pesticides are known to be used worldwide to control certain bee diseases and pests in apiculture. However, in most instances, their handling and administration are uncontrolled and can be applied without approved protocols.

The use of such chemicals inside a beehive can therefore cause direct contamination of honey. Moreover, use of pesticides in agriculture is a common practice to increase productivity. Therefore, pesticides’ residues detected include acaricides, organic acids, insecticides, fungicides, herbicides, and bactericides (38). In addition, non-respect of good phytosanitary practices can cause contamination to the environment, animals, and humans. Apiarists make use of antibiotics in the hive to treat bacterial diseases. As a result, traces of drugs can be found in the honey itself. Residues of oxytetracycline and chloramphenicol have been found above accepted regulatory standards set for honey (39). Same authors indicate that other antibiotics are also used: erythromycin, lincomycin, monensin, streptomycin, and enrofloxacin. Presence of antibiotics’ residues is most often the result of improper management and bad beekeeping practices. Drugs’ residues have already been found to be above regulatory standards (39).

In 2007, Tchoumboue et al. determined characteristics (physicochemical and microbiological) of honey collected from the West region (Sudano-Guinean zone). They bought 43 honey samples from the local markets and directly collected 7 additional samples from the bee research farm of the University of Dschang to be used as reference honey sample. More than 73.47% of honey samples bought in local markets were also contaminated with microbes (*Bacillus* sp. and fungi). The most frequent fungi in decreasing order were *Candida, Aspergillus, Geotrichum*, and *Rhizopus* spp. Important sources of contamination are handling and adulterations, as confirmed by the absence of associated contamination in the honey harvested in bee farms where processing and handling are carried out in better hygienic conditions (40).

### Insects

Insect consumption is widespread in Cameroon. A lot of studies have demonstrated that edible insects contain important levels of good quality and highly digestible proteins (41, 42). Insects are also rich sources of fat, vitamins, and minerals, in particular iron and zinc (43–45). Commonly consumed insects include termites, locusts, grasshoppers, weevils, and caterpillars (46). Examples of toxic insects are given, but often traditional cooking methods are used to remove the poisoning substance. Eating insects does not depend only on taste and nutritional value but also on cultural considerations (customs, ethnic preferences, or prohibitions) (42). Culinary treatment in which these insects undergo before consumption varies from one ethnic group to another. Regarding locusts, they are eaten raw within certain ethnic groups or they are boiled, smoked, fried in oil before consumption. In all cases, insect consumption may prove to be dangerous to human health. According to European Food Safety Agency, there are possibilities for transmission of various contaminants (chemical, microbiological, etc.) on insects during their nutrition. For example, Ene indicates that the occurrence of prions in non-processed insects is related to whether or not the substrate includes protein of human origin or ruminant origin. Some authors conclude that environment and production, as well as the substrate in use, the stage and period of harvest, and the insect species can have important impact on the occurrence of chemical and biological hazards in foods and feeds derived from insects. Therefore, related environmental hazards are expected to be comparable to other animal’s production systems (47).

### Feedstuffs

In Cameroon, animal feed production remains artisanal. The first national survey of animal feed factories was done in 2014 by the Ministry of Fishery, Livestock and Animal Industries (MINEPIA). This survey aimed at making an appraisal diagnostic situation of the sector. Six out of 10 regions were included for their importance in the production of at least one of the ingredients of the feeds. West, Littoral, and Center Regions represented 85% of total production (48). Traditional poultry production is the most important production systems. Chickens, pigs, ducks, and pigeons are the dominating produced species. MINEPIA (49) identified four types of feed in some rural farms in Bamenda (North-west region). Most often, feed factories proposed cereals based feeds composed usually of maize, soya beans, fish flour, minerals, concentrates, vitamins, additives, and bone powders. Feed ingredients are purchased locally or imported from various countries, and mixed together in specific proportions using artisanal grinder (50). Feed factories surveyed in Cameroon have been found to work in unhygienic conditions, thus rendering animal feeds a possible vector of toxicants. In the farm-to-fork model, animal feeds are known to be at the beginning of the food safety chain. Animal feeds are frequently contaminated by bacterial foodborne pathogens like Non-Typhi serotypes of *Salmonella enterica* (51), fungi species *Aspergillus flavus, Aspergillus niger, Aspergillus oryzae, Fusarium solani, Fusarium verticillioides, Penicillium* spp., and *Rhizopus* spp. (52, 53). Maize grains that are spoiled and different types of snacks that are consumed in the Western Highlands of Cameroon have been found to be infected by several mycotoxin producing fungi. *Fusarium* and *Aspergillus* species were isolated in the frequency ranging from 20 to 100% presence in the samples analyzed, while *Staphylococcus* and *Salmonella* species were the most isolated bacteria (54). These fungi (*Fusarium* and *Aspergillus* species) in certain conditions can produce toxic metabolites and mycotoxins. For instance, the presence of ochratoxin A in foods of animal origin may occur as a result of direct fungi contamination or indirectly *via* contaminated feeds (55, 56). The cases of fumonisins, B-trichothecenes, zearalenone, fumonisins, aflatoxins and ochratoxin A (56); the case of fumonisins, deoxynivalenol, and zearalenone have also been detected in maize sampled in Cameroon (54). Farmers and traders adopted some practices that exposed cereals grains and other feeds to mycotoxins contamination. Rodrigues et al. in 2011 underlined on (i) the use of stock seed as planting materials by farmer, (ii) delayed harvesting, (iii) heaping of harvested maize cobs on the field, (iv) broadcasting method use for planting, (v) dipping and teeth cracking method with hand to determine dryness of maize, (vi) use of wooden stalls with poor ventilation for maize storage at market centers, and (vii) temporal storage in the open air, resulting in moisture re-absorption (57). Some of these feeds contaminants are of great public health concerns, although they remain ignored and unaddressed in some countries (58). Prevalence of animal feed contamination by mycotoxins is frequently high. Kana et al. (54) sampled 201 farms products (maize, crab peanuts, poultry feed) in three different agro-ecological zones in Cameroon. They detected aflatoxins in 9% of maize samples, 100% of crab peanuts, and 93.3% of poultry feeds. There were no significant differences in the level of contaminations across all three agro-ecological zones. In a similar study, Abia et al. (59) sampled 20 poultry feeds in different farms and analyzed them for 320 fungi metabolites. Deoxynivalenol and fumonisins were dominants in samples from the West Region of Cameroon, while aflatoxins were dominants in sample from Yaounde. Average aflatoxin B1 concentration (40 µg/kg) was higher than the European Union, Codex Alimentarius, China, and USA tolerable limits. Ediage et al. (60) analyzed 420 food items (maize, peanuts, and cassava) from three agro-ecological zones and tested for the presence of 25 mycotoxins: 51% of all samples were positives to at least one mycotoxin, 74% for maize, 62% for peanuts, and 24% for manioc. Aflatoxin prevalence for all samples was 22%. Moreover, zearalenone were detected in 14% of the maize samples, but all concentrations were below the European Union tolerated maximum level in non-processed maize products (350 µg/kg). Since several deaths of children in Africa are suspected to be caused by mycotoxins compounds (61, 62), the issue deserve serious assessment and management.

## The Legal Operating Framework

Food control and inspections are governed by laws, and regulations competent authorities elaborate rules and standards, and then ensure enforcement. These regulations have to define the principle and scope of the law, as well as the roles of each party.

In Cameroon, a specific food law does not exist. Control of food industries is regulated by the law no. 98/015 of July 1998 on hazardous food settlements that categorize operators, modalities for inspections, and responsibilities of each party engaged in the process. This law embodies all the activities of the economic sector and are not specific to agricultural and food industries. Many others regulations are then taken from other administrations and agencies to ensure its implementation. The Prime Ministerial order no. 99/918 PM of November 1999 defined the modalities for the exploitation of hazardous settlements, including agricultural and food industries. Another order (no. 2012/382 of September 2012) creating and organizing the MINEPIA conferred to this ministry the elaboration of government policies concerning issues on food of animal origin (agreement and authorization, promotion of hygiene in animal industries, law enforcement, standards elaboration). MINEPIA is therefore responsible only for certain sectors of the food chain from farm-to-fork. This situation is also true for other administrations. Consequently, Cameroon experienced non-coordinated actions, overlapping between many actors. The Prime Minister order no. 2014/2379 PM of August 20th 2014 set modalities for the coordination of inspections and official control of enterprises susceptible to generate risks for workers and population. These legal dispositions are completed by certain standards already homologated at national level to serve as a guide. More than 20 standards concerning food of animal origin exist, with a good number of them transformed as technical regulation, to enforce and ease official control activities at the national level. Surveillance and quality assessment of these products is becoming an urgent issue for population health. The creation of a toxicovigilance system as described by Pouokam et al. (63) is a crucial step in ensuring the wholesomeness of foods of animal origin in Cameroon. Besides, setting of technical standards for periodic controls will help improve the overall quality of meat food. Since 2002, a laboratory for analysis of foods of animal origin has been constructed and partially equipped within the MINEPIA in Douala, but unfortunately it is not yet functional till date.

## One Health: Conclusion and Perspectives

Foods of animal origin eaten by the Cameroonian population are found to be contaminated by microbial and chemical contaminants, and most often by a mixture of both categories (see Table 1).

**Table 1:** Summary on the contamination of some foods of animal origin in Cameroon.

Food items	Contaminant risks	Reference
Fish	Mercury	(12, 14)
	Aluminum, cadmium, lead	(12)
Smoked fish	Toxics products used for cashing and smoking	(15)
Smoked fish	*Escherichia coli*, fecal streptococci, *Staphylococcus aureus*, sulfite-reducing clostridia, and molds	(16)
Smoked fish and shrimps	Lead, nickel	(12)
Outdoors meals	Methylmercury	(18)
Fish	Pesticides residues	(19)
Frozen chicken	*E. coli, Campylobacter*, and *Salmonella*	(20)
Gizzard and chicken muscle	Aflatoxin B1	(21)
Poultry meat	Cadmium	(22)
Eggs	Aflatoxins B1 and B2, cadmium	(22)
Beef meat carcasses in slaughterhouses	Mesophilic aerobic bacteria, coagulase-positive staphylococci, anaerobic sulfur-reducing bacteria, thermo tolerant coliforms	
“*Kilishi*” (dried meat)	Bacterial, mold, and yeast	(26)
Pork meat (street vended)	*S. aureus, Klebsiella pneumoniae, Escherichia coli, Salmonella* spp., *Proteus vulgaris*, and *Shigella* spp.	(27)
Street-vended meat (roasted beef meat, fried pork meat, and roasted chicken)	*S. aureus, Bacillus cereus, Salmonella, E. coli* type 01 non-0157:H + *E. coli* strain, yeast, and molds	(28)
*Lebol* (traditional fermented milk), *Kindirmou* (traditional butter)	Yeast and molds	(29)
Raw milk (cow)	Aflatoxin M1 and penicillin, oxytetracyclin, streptomycin	(23, 32)
Honey	Pesticides residues and residues of oxytetracycline and chloramphenicol	(38, 39)
Honey	*Candida, Aspergillus, Geotrichum*, and *Rhizopus* spp.	(40)
Insects	Prions	(47)
Feedstuffs	Non-Typhi serotypes of *Salmonella enterica* *Aspergillus flavus, Aspergillus niger, Aspergillus oryzae, Fusarium solani, Fusarium verticillioides, Penicillium* spp., and *Rhizopus* spp. Myctoxins (aflatoxin, deoxynivalenol, fumonisins, zearalenone)	(51) (52, 53) (59, 64)

More often, the concentration of contaminants varies with agro-ecological zones, harvesting seasons, preparation, and cooking methods (63). Compared to existing international norms, some of these contaminants exceed the legal maximum or tolerable limits. Disease risks are linked to the level of exposure to these contaminants. The association between exposure to contaminants and prevalence of certain diseases among the population remains critical but often difficult to establish. Proietti et al. (65) underlined the need to consider cultural behaviors in building reliable exposure scenarios to appreciate the level of health risk. In the first report on global burden of foodborne diseases, the WHO estimates the disease adjusted life years (DALYs) of some selected food hazards. Thirty one foodborne hazards, found to cause 32 diseases, were identified and included in the study. Examples of included hazards are aflatoxin, peanut allergens, dioxin, and cyanide in cassava. In that study, disease burden due to aflatoxins was estimated using a counterfactual approach, i.e. by estimating i) the relevant diseases fraction via the exposure estimate, ii) the carcinogenicity potency factors, and iii) by applying these factors to WHO estimates for incidence and mortality using the case of hepatocellular carcinoma. Forty % of the foodborne disease burden was recorded among children less than 5 years of age worldwide; with 18 million DALYs attributed to foodborne diarrheal disease agents. The highest burden per population was observed in Africa (60). Poor hygienic working environment and lack of official control along the food chain is an aggravating factor for contamination. Pouokam (51) audited some animal feed factories in Yaounde to assess their conformity to good hygienic practices. All feed factories failed. Their working conditions revealed a lot of weaknesses and absence a food management system. The actual legal and technical framework does not allow products surveillance and inspection to be done properly (66). With the creation of the national quality and standards agency, certain norms have been approved and transformed into technical regulations for enforcement. Unfortunately, the Ministry in charge of foods inspection is not yet fully operational. Some data on foods contamination are produced in various university laboratories and research institutions, but the absence of a coordinating body led to an under-exploitation of existing data in policy formulation. A National Public Health Observatory exists at the Ministry of Public Health, with a mandate that could allow for the overseeing of these activities, but is not yet fully operational. Another body that could be suitable to take over these actions is a national One Health program.

**Figure 2:**
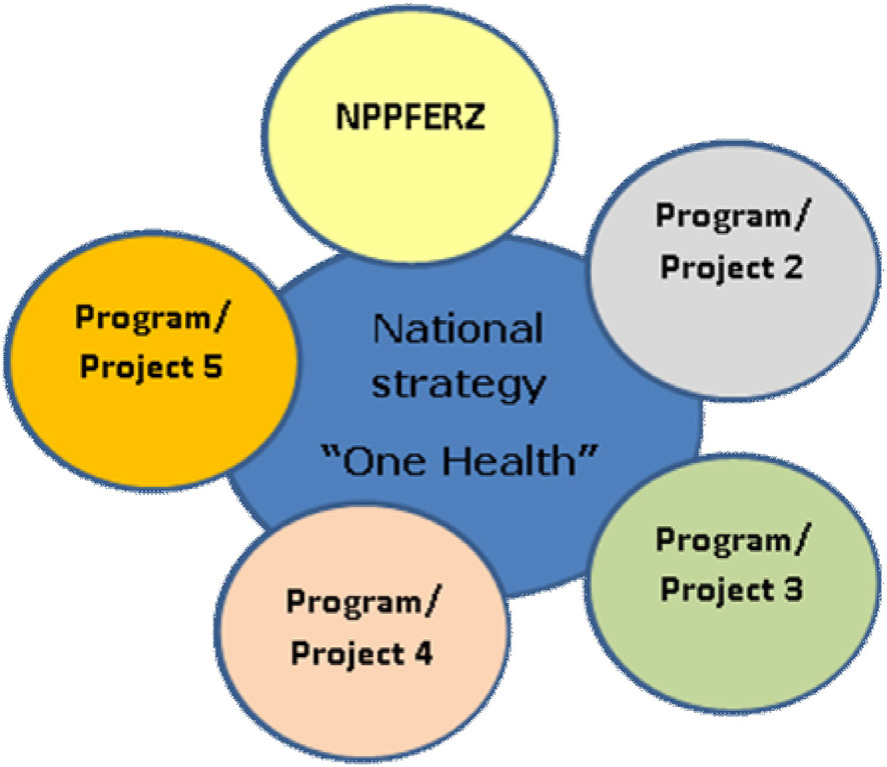
Cameroon One Health strategy components. NPPFERZ, National Program for the Prevention and Control of Emerging Re-emerging Zoonoses.

One Health, i.e. a science-based approach linking human health and nutrition with animal and environmental health, calls for improved collective and concerted actions across the three sectors (environment, animal, and human). Operationalizing this concept in complex health challenges like food safety requires building first on the global institutional framework (67). For instance, this calls for changes in the ongoing models of training and implementation of public health policies in African countries (68). These changes pivot on improved stakeholders’ perception of implication of their work on public health as well as the identification of both actors (from field production of raw materials, to management and policy) and interactions and dynamics among them. A One Health working framework can provide an integrated food safety risks understanding and management, from the whole ecosystem of the food system by using a web of causation approach (69, 70). The first One Health workshop was organized by Cameroon in 2011 with all stakeholders to define the national One Health strategies. Today, a coordination structure under the supervision of the Prime Minister’s office has been put in place. This position helps to speed up the decision process and ensure full participation of stakeholders. There are regular meeting sessions between members of the committee including laboratories, universities, training schools, and ministries. The Cameroonian One Health strategy was launched in 2013 with the development of the program for the prevention and control of zoonotic diseases. Today, the Committee has successfully delivered two documents: the National One Health Strategy (Figure 2) chaired by the Prime Minister with 11 ministers as members and the National Program for the Prevention and Control of Emerging and Re-emerging Zoonosis, which is part of the implementation of the One Health strategy.

The ongoing program covers the surveillance of diseases in wildlife, prevention and control of rabies, capacity building for the detection and risk analysis of zoonoses, and integrated rapid responses systems. The program does not take into account zoonosis from feeds and foods of animal origin, nor toxic chemicals that can both be transferred from feed and food of animal origin to human beings and from mother to the child, thus constituting “toxicant-related zoonosis” as described by Frazzoli and Mantovani (71) and Frazzoli, Bocca, and Mantovani (72). Therefore, programs for feed and food surveillance need to be established with a more integrated understanding on the transfer and circulation of harmful microbial and chemicals agents across the three components of the One Health web (environment, animals and humans).

The One Health committee should shift from an administrative tool to a more science-based and technical body, in charge of assessing, planning, and centralizing each stakeholder contributions from the three components geared at addressing food safety risk in the entire farm-to-fork chain. Finally, Frazzoli et al. about the concept of sustainable food safety (SFS), define it as the complex of actions to “build” the healthy growth and adulthood of the new generation through proper and safe nutrition *in utero* and in early years of life (67). In this paradigm, the need for actions appears urgent in developing countries, such as Cameroon, where growth problems and preventable morbidity and mortality are still high in newborns and young children (70). As illustrative scenario, in many Cameroonian communities the whole family eats the same meal from a common pot. “Special” recipes for young children, pregnant, and/or breastfeeding mothers are not envisaged within the local eating culture. Thus, the SFS framework should address widely and highly consumed ingredients of main daily traditional recipes and diets, while nutrition of fetuses and newborns depend on the maternal diet (transgenerational diet) during both pregnancy and breastfeeding.

Africa is an emerging food producing area and aspects should be examined, namely: (i) the farming scenario and its environment; (ii) primary production role in food security and safety; (iii) risk management pillars as modern infrastructures, effective farmer organizations, and institutional systems to guarantee animal health and safety of products; (iv) feasible interventions to protect food chains from hazards (e.g., sustainable use of fertilizers, feeds, veterinary drugs, pesticides) at farmers’ community level, based on good practices and risk assessment; and (v) transnational consortium as a platform for technology transfer and solution exchange (63, 70, 71, 73).

Social innovation based on the empowerment of the primary food producers emerges as crucial for sustainable and safe food production (74). Sustainable policies should be supported by the mobilization of stakeholders of One Health (35, 74).

Poverty and inequality underlie high rates of communicable diseases, and also give rise to NCD risk factors including poor and unsafe diet, driving a double burden of disease, particularly among rural communities and infants, requiring a Global Health action (75).

## Conclusion and Recommendations

Foods of animal origins constitute an important share of Cameroonian diet. Smoked and fresh fish, poultry, pork and beef meats, eggs, milk and dairy products, shrimps, honey, and insects make up the top foods items consumed between 1.3 and 22.4 g/day. Contaminants analysed and found in these food items are toxic metals (aluminum, cadmium, lead, arsenic, methylmercury, vanadium), mycotoxins (aflatoxins), veterinary drugs’ residues (oxytetracyclin, streptomycin, penicillin), pesticides, and microorganisms (*Salmonella* sp., *Campylobacter* sp., *E. coli, S. aureus, Bacillus* sp.). Efforts made so far by authorities to guarantee the safety of foods remain largely ineffective and inefficient, exposing populations to hazards with potential huge health impacts. This review can serve as an initial step to evaluate and document specifics risks scenarios, as well as short- and long-term preventive actions to mitigate risks. For this purpose, the One Health approach appears as an appropriate tool to carry out situational and integrated diagnostic risk assessments studies.

## Author Contributions

All authors listed have made a substantial, direct, and intellectual contribution to the work and approved it for publication.

## Conflict of Interest Statement

The authors declare that the research was conducted in the absence of any commercial or financial relationships that could be construed as a potential conflict of interest.
